# Clinical relationship between auditory neuropathy and nervous system diseases

**DOI:** 10.12669/pjms.336.13225

**Published:** 2017

**Authors:** Jingbo Wang, Lanlan Jin, Jun Chen, Xiaobi Fang, Zhisu Liao

**Affiliations:** 1 Jingbo Wang, Department of Otorhinolaryngology, The First Affiliated Hospital of Wenzhou Medical University, Wenzhou 325000, P. R. China; 2 Lanlan Jin, Department of Otorhinolaryngology, The First Affiliated Hospital of Wenzhou Medical University, Wenzhou 325000, P. R. China; 3 Jun Chen, Department of Otorhinolaryngology, The First Affiliated Hospital of Wenzhou Medical University, Wenzhou 325000, P. R. China; 4 Xiaobi Fang, Department of Otorhinolaryngology, The First Affiliated Hospital of Wenzhou Medical University, Wenzhou 325000, P. R. China; 5 Zhisu Liao, Department of Otorhinolaryngology, The First Affiliated Hospital of Wenzhou Medical University, Wenzhou 325000, P. R. China

**Keywords:** Auditory neuropathy, Pure-tone hearing threshold, Acoustic immittance, Auditory brainstem response, Vestibular function, Nervous system disease

## Abstract

**Objective:**

To explore the clinical relationship between auditory neuropathy (AN) and nervous system diseases.

**Methods:**

A total of 134 AN patients who were treated in our hospital from December 2011 to April 2016 were selected. Then 120 cases (240 ears) with complete data of pure tone audiometry and acoustic immittance test were selected as an AN1 group, which was compared with 30 patients (49 ears) with general sensorineural hearing loss (SHL) in regard to the results of pure tone audiometry and acoustic immittance test. On the other hand, 79 cases (158 ears) of the 134 patients with complete data of DP otoacoustic emission test were selected as an AN2 group, which was compared with 30 normal subjects (60 ears) regarding the results of DP otoacoustic emission test.

**Results:**

Increases in the pure-tone hearing threshold by air conduction of AN1 group significantly exceeded those of SHL group at 0.125 and 0.25 kHz (low frequency) (P<0.05). The former group had significantly lower values at 1.0, 2.0 kHz (moderate frequency) and 4.0, 8.0 kHz (high frequency) (P<0.05). Of 134 patients, 14 (19 ears) had evoked V wave upon auditory brainstem response, whereas no waves after I wave were evoked in other tested ears. Distortion product (DP) otoacoustic emissions could all be evoked. AN2 group had significantly higher amplitudes of DP-gram than those of normal control group at 0.5 and 0.7 kHz (low frequency) (P<0.05). Except for three cases of unsteady walking and 10 of dizziness, others did not suffer from typical symptoms of vertigo attack. As to caloric test-induced electronystagmograms, there were 30 bilaterally normal cases (75.0%), one case of left-side semicircular canal paresis (25%) and nine cases of bilateral semicircular canal paresis (22.5%). Four patients with other nervous system diseases were complicated with AN. Other nervous system disorders included three cases of optic nerve atrophy and 7 of lower limb nerve damage.

**Conclusion:**

According to characteristic hearing dysfunction, AN may occur in the afferent pathway of acoustic nerve, probably accompanied by the pathological changes of efferent nerve in the olivocochlear system inside the brainstem.

## INTRODUCTION

First named by Starr et al. in 1996, auditory neuropathy (AN) is a sensory neurological hearing loss with special clinical manifestations induced by the damage of auditory nerve branch of the VIII cranial nerve.^1^ Its audiological test results are usually inconsistent. AN is mostly typified as missing or seriously abnormal auditory brainstem response and normal evoked otoacoustic emission.^2^

Otoacoustic emission provides reliable evidence for the identification and diagnosis of cochlear and retrocochlear lesions.^3^ Despite numerous studies on AN, there remains controversy over its pathological sites, etiology, pathogenesis and epidemiological characteristics, and effective therapies are still lacking.^4^ Therefore, this still needs to be studied further.

In this study, we selected AN patients and those complicated with nervous system diseases, and performed clinical investigation and audiological examination. The aims of this study were to summarize the clinical manifestations and audiological characteristics of AN, to explore the roles of pure tone audiometry, acoustic immittance test, auditory brainstem response test and distortion product (DP) otoacoustic emission test in AN diagnosis, to investigate whether AN was complicated with vestibular function disorders or other nervous system diseases, and importantly, to provide valuable evidence for future clinical and experimental studies on AN.

## METHODS

This study has been approved by the ethics committee of our hospital, and written informed consent has been obtained from all patients. A total of 134 AN patients who were treated in our hospital from December 2011 to April 2016 were selected, including 48 males and 86 females. They first visited hospital at 1/6-48 years old, with the average of (20.0±8.45). The patients first had symptoms from newly born to 39 years old, with the average of (17.1±7.89). The disease courses ranged from 1/6 to 15 years, with the average of (3.7±4.87). All patients had normal bilateral eardrums. CT and/or MRI did not disclose any abnormalities. We excluded external ear, middle ear and other ear diseases as well as systemic diseases that may affect hearing, such as otitis externa, impacted cerumen, otitis media, noise damage, ototoxic drug poisoning, inner ear malformation, diabetes mellitus, hypertension and chronic nephritis.

### Subjects and grouping

Of the 134 enrolled patients, 120 cases (240 ears) with complete data of pure tone audiometry and acoustic immittance test were selected as an AN1 group. Meanwhile, 30 cases (49 ears) of sensorineural hearing loss (SHL) were selected as a control group. There were 11 males and 19 females aged 11-45 years old, with the average of (26.8±10.24). The two groups had similar age and gender ratio. The two groups were compared as regard the results of pure tone audiometry and acoustic immittance test.

A total of 79 cases (158 ears) with complete data of DP otoacoustic emission test were selected as an AN2 group. Another 30 normal young subjects (60 ears) consisting of 12 males and 18 females aged 12-25 years old (average: 20) were selected as a normal control group. The air-bone conductions of both ears in pure tone audiometry were lower than 25 dB HL (0.125-8 kHz). Acoustic immittance test showed A-shaped tympanometry curves of both ears as well as normal acoustic reflex thresholds of ipsilateral and crossed stapedius muscles. The two groups had similar age and gender ratio. The two groups were compared regarding the results of DP otoacoustic emission test.

Of the 134 AN patients, 40 were complicated with vestibular function disorders, 4 were complicated with nervous system diseases, and 19 were complicated with other nervous system disorders.

### Pure tone audiometry

Air-bone conductions in pure tone audiometry at seven frequencies were detected from 125 to 8000 Hz by GSI 61 pure tone audiometer according to China National Standard GB7583-87.

### Acoustic immittance test

Tympanometry curves were plotted by GSI 33 middle ear analyzer. Acoustic compliances of both ears and acoustic reflex thresholds of ipsilateral and crossed stapedius muscles at 1, 2 and 4 kHz were measured, and those exceeding 110 dB HL were considered unevoked.

### Auditory brainstem response test

This test was conducted by using Spirit Evoked Potentials System (Nicolet) with silver plate electrodes. A recording electrode was placed at the Cz point or in the middle of forehead close to the hair line, and a reference electrode was placed at the ipsilateral mastoid. Acoustic stimuli were clicked with the maximum output sound intensity of 103 dB nHL (0 dB nHL=30 dB peSPL), scanning time of 20 ms, filter range of 100-300 Hz, stimulus frequency of 10 Hz and superposition of 1024 times. The wave mode and central conduction time were recorded.

### DP otoacoustic emission test

This test was carried out with HIS Version3.2 acoustic emission detector (USA). To ensure the external auditory canal was completely sealed during test, ER-10C probe was connected to a proper rubber earplug and then with amplifier and DSP plate through wires for operation. The patients were tested in a soundproof room with <40 dB SPL noise. In the sitting position, patients were required to keep sober, quiet and not to swallow. Wires were fixed by small clamps, without contacting the body. The ratio of original stimulus frequencies f2/f1 was 1.22, and f0=(f1×f2)^1/2^, with the signal-to-noise ratio of 3 dB. The results at each frequency were superposed 24 times. DP otoacoustic emission was detected at f0 of 0.5, 0.7, 1.0, 1.4, 2.0, 3.0, 4.0, 6.0 and 8.0 kHz as well as at the stimulus intensities of L1=70 dB SPL and L2=65 dB SPL respectively. Using amplitude of DP otoacoustic emission test and background noise as the y axis and f0 as the x axis, corresponding curves were plotted. Twenty AN patients were subjected to DP acoustic emission test in the presence or absence of contralateral sound stimulation that was continuously created by pure tone audiometer at the wideband white noise of 65 dB SPL with TDH39 earphone.

### Test of AN complicated with vestibular function disorders

Nieolet Nystar™ Plus electronystagmograph (USA) was employed to record electronystagmograms and to perform vestibular function examinations. Caloric test was carried out using the Hallpike method with maximum slow phase velocity (MSPV) as the index. According to the Jongkee’s formula, semicircular canal paralysis (Cp) and directional preponderance (Dp) were calculated. The results were analyzed according to the determination criteria of Samaha et al.^5^ MSPV values of each caloric test for both ears were ≤8 degrees per second or the total MSPV of four stimulations was <30 degrees per second were/was determined as reduced bilateral semicircular canal reactions. MSPV values of two caloric tests for one ear were ≤8 degrees per second or DP of the contralateral ear was ≥20% were/was determined as reduced unilateral semicircular canal reactions. The others were normal bilateral semicircular canal reactions.

### Test of AN complicated with nervous system diseases

All patients were subjected to disease history inquiry, comprehensive nervous system examination, electromyography, nerve conduction velocity test and immunological examination of cerebrospinal fluid. In addition, they received pure tone audiometry, acoustic immittance test, DP otoacoustic emission test and auditory brainstem response test. The patients with dizziness or extremely severe deafness received vestibular function examination and electronystagmography, and those with visual loss underwent visual electrophysiological examination. The imaging examinations included temporal bone thin-section CT and/or cranial MRI.

### Statistical analysis

All data were analyzed by SPSS for Windows. The same variables of two groups were subjected to independent samples t-test, and those with variance heterogeneity were compared by t test. P<0.05 was considered statistically significant.

## RESULTS

### Pure tone audiometry results

The air-bone conductions of both groups decreased. In the AN1 group, 167 (69.6%), 45 (18.8%), 23 (9.6%) and 5 (2.1%) ears had rising, trough, flat and falling curves respectively. According to the average pure-tone hearing threshold by air conduction at 0.125-0.5 kHz (low frequency), 17 cases had mild hearing disorder (26-40 dB HL, 14.2%), 35 had moderate hearing disorder (41-55 dB HL, 29.2%), 42 had moderate-to-severe hearing disorder (56-70 dB HL, 35.0%), 20 had severe hearing disorder (71-90 dB HL, 16.6%) and six had extremely severe hearing disorder (>90 dB HL, 5.0%).

In the SHL group, four, eight and 37 ears had trough, flat and falling curves respectively. There were 3 cases of mild hearing disorder (26-40 dB HL, 6.1%), 13 of moderate hearing disorder (41-55 dB HL, 26.5%), 20 of moderate-to-severe hearing disorder (56-70 dB HL, 40.8%), nine of severe hearing disorder (71-90 dB HL, 18.4%) and four of extremely severe hearing disorder (>90 dB HL, 8.2%).

Increases in the pure-tone hearing threshold by air conduction of the AN1 group were significantly higher than those of the SHL group at 0.125 and 0.25 kHz (P<0.05). However, the former group had significantly lower values at 1.0, 2.0 kHz (moderate frequency) and 4.0, 8.0 kHz (P<0.05) (Table-I).

**Table-I:** Increases in the pure-tone hearing threshold by air conduction.

*Group*	*Ear*	*Frequency (kHz)*						

		*0.125*	*0.25*	*0.5*	*1.0*	*2.0*	*4.0*	*8.0*
SHL	49	54.5±21.8	56.3±23.1	61.3±23.7	62.0±24.7	67.3±23.8	81.3±21.2	85.3±21.0
AN1	240	61.3±19.7*	64.1±20.2*	59.5±20.6	49.5±22.9*	40.1±25.4*	38.7±255*	45.6±26.3*

* Compared with SHL group, P<0.05.

### Acoustic immittance test results

In the AN1 group, seven cases (8 ears) and eight cases (12 ears) had A_D_- and A_S_-shaped tympanograms respectively, and the other 220 ears all showed A-shaped curves. The static compliance values were 0.2-2.2 ml. No acoustic reflexes of ipsilateral or crossed stapedius muscles in 63 cases were unevoked. Although acoustic reflexes at single or multiple frequencies were evoked from 57 cases (97 ears; 40 cases of bilateral and 17 of ipsilateral), the muscle reflex thresholds all increased. All ears were free from loudness recruitment. In the SHL group, one case (1 ear) had A_S_-shaped tympanogram, and the other 48 ears all showed A-shaped curves. The static compliance values were 0.2-1.4 ml. No acoustic reflexes of ipsilateral or crossed stapedius muscles in 11 cases (18 ears) were unevoked. Acoustic reflexes at single or multiple frequencies were evoked from 19 cases (31 ears), and 9 ears suffered from loudness recruitment.

### Auditory brainstem response test results

Of the 134 AN patients, 15 (19 ears; 4 cases of two ears and 11 of one ear) had evoked V wave upon auditory brainstem response, with the thresholds of 30-90 dB SPL.I wave could be evoked from the left ear of one patient. No waves after I wave (>100 dB SPL) were evoked in other tested ears.

### DP otoacoustic emission test results

DP otoacoustic emissions could all be evoked. The AN2 group had significantly higher amplitudes of DP-gram than those of the normal control group at 0.5 and 0.7 kHz (low frequency) (P<0.05). Nevertheless, the two groups had similar results at 1.0-2.0 kHz (moderate frequency) and 3.0-8.0 kHz (high frequency) (P>0.05) (Table-II). The difference between DP-gram amplitude and background noise of the AN2 group, which was significantly higher than that of the normal control group at 0.7 kHz, was significantly lower at 3.0 kHz (P<0.05). The differences were not significant at other frequencies (P>0.05) (Table-III). With and without contralateral acoustic stimuli, 20 AN patients (40 ears) had similar amplitudes of DP-gram at 9 tested frequencies (P>0.10) (Table-IV).

**Table-II:** DP-gram amplitudes.

*Group*	*Ear*	*Frequency (kHz)*								

		*0.5*	*0.7*	*1.0*	*1.4*	*2.0*	*3.0*	*4.0*	*5.0*	*6.0*
Normal control	60	8.0±5.0	8.8±4.7	11.8±5.8	12.7±6.2	10.5±6.2	7.7±5.5	6.8±4.4	5.3±5.5	2.4±6.6
AN2	158	10.1±5.1*	12.9±5.1*	13.2±6.5	14.2±6.5	9.7±5.9	6.0±6.0	5.6±6.5	5.1±7.2	2.5±7.4

* Compared with normal control group, P<0.05.

**Table-III:** Differences between DP-gram amplitude and background noise.

*Group*	*Ear*	*Frequency (kHz)*								

		*0.5*	*0.7*	*1.0*	*1.4*	*2.0*	*3.0*	*4.0*	*5.0*	*6.0*
Normal control	60	9.0±6.0	12.0±6.6	18.6±6.7	20.7±6.7	18.5±6.9	17.4±5.6	16.1±4.6	14.8±5.5	12.0±6.6
AN2	158	9.3±5.6	14.7±6.7*	17.5±7.3	20.7±6.9	17.7±5.7	15.1±6.2*	15.4±6.5	14.0±7.1	11.2±7.4

* Compared with normal control group, P<0.05.

**Table-IV:** DP-gram amplitudes with and without contralateral acoustic stimuli.

*Group*	*Frequency (kHz)*								

	*0.5*	*0.7*	*1.0*	*1.4*	*2.0*	*3.0*	*4.0*	*5.0*	*6.0*
With stimuli	9.2±4.5	12.5±5.1	12.1±5.3	13.4±6.1	7.9±6.4	5.5±5.3	4.9±4.6	2.9±7.4	-0.4±7.4
Without stimuli	9.7±5.0	12.0±5.9	12.4±5.6	13.6±5.9	7.7±6.2	5.6±6.0	4.4±5.0	2.9±77	-0.4±7.8
P value	0.452	0.357	0.433	0.627	0.716	0.863	0.310	0.966	0.937

### Test results of AN complicated with vestibular function disorders

Except for three cases of unsteady walking and 10 of dizziness, others of the 40 patients did not suffer from typical symptoms of vertigo attack such as illusion of rotation, nausea and vomiting. Of the three cases of unsteady walking, one had left-side semicircular canal paresis, 1 had bilateral semicircular canal paresis and another who had normal vestibular function was complicated with severe visual loss. Of the 10 cases of dizziness, 2 had normal vestibular function, 5 had bilateral semicircular canal paresis, one had left-side semicircular canal paresis and two had evoked positional nystagmus. All patients had normal optokinetic test results, with Type-I and II visual tracking curves. As to caloric test-induced electronystagmograms, there were 30 bilaterally normal cases (75.0%), one case of left-side semicircular canal paresis (25%) and nine cases of bilateral semicircular canal paresis (22.5%). The average ages of 30 cases with normal vestibular functions and 10 cases with abnormal ones were (22.0±6.73) and (20.3±7.97) years old respectively. Another two cases suffered from evoked positional nystagmus, of whom one had rightward horizontal spontaneous nystagmus, with normal electronystagmograms though.

### Test results of AN complicated with nervous system diseases

Four AN patients were complicated with other nervous system diseases, i.e. 1 case of progressive papillary muscle atrophy, two cases of Friedreich ataxia and 1 case of Refsum disease. Other nervous system disorders included three cases of optic nerve atrophy, 6 cases of vestibular neuropathy and 7 of lower limb nerve damage.

## DISCUSSION

For SHL, hair cells in the cochlear high frequency region are more easily involved, often showing a descending hearing.^6^ SHL based on low frequency hearing loss can also be seen in the early Meniere’s disease. Due to loudness recruitment, stapes muscle reflex can often be induced, the low frequency amplitude of distortion product otoacoustic emission is decreased, auditory brainstem response wave I and action potential can be induced, and patients have a typical triple sign, i.e. repeated episodes of vertigo, deafness and tinnitus.^7^

The tympanogram of sensorineural deafness also showed “A” type curve, the stapedius muscle acoustic reflex threshold could also be increased or not induced, but some patients with SHL might have loudness recruitment. The mechanism of loudness recruitment is not yet clear, but it is common in general sensorineural deafness and considered an indication of cochlear hair cell diseases.^8^ All AN patients did not show loudness recruitment, indicating that the auditory nerve transmission pathway of AN was affected.

Starr et al. suggested that the lesions of AN lied in the VIII brain auditory branch before the cochlea entering the brainstem, including: inner hair cells, synaptic connections between inner hair cells and VIII brain the nerve fibers, spiral ganglion, cochlear nerve fibers, VIII brain nerve and its part associated with the above sites, but they did not include brainstem auditory pathway lesions.^1^ In this study, the auditory brainstem response of all AN patients from wave I was not induced (>l00 dB SPL), except that wave V was induced from one ear of one patient, and from 19 ears of 14 patients. Auditory brainstem response wave I was mainly sourced from the cochlear afferent nerve,^9^ and the auditory brainstem response missed from wave I, suggesting that the lesions of auditory branch and/or inner hair cells, inner hair cells and afferent synapses of VIII brain nerve leads to unsynchronized nervous discharge of the nerve fibers. As there are no methods checking the function of hair cells within the cochlea in current clinical practices, the functional status of inner hair cells cannot be correctly evaluated yet. AN is generally involved the bilateral auditory nerves.^10^ Herein, only one patient had normal auditory brainstem response in the left ear and AN in the right ear according to the hearing test results. Whether a patient with unilateral AN can eventually develop into bilateral AN still needs further observation and follow-up. Mohammadi et al. also reported unilateral AN, similar to this study.^11^

Ten patients with vestibular dysfunction herein had an average age of 20.3 years old, and that of 30 patients with normal vestibular function was 22.0. AN complicated with vestibular dysfunction had no obvious correlation with age. It is necessary to observe the changes of vestibular function in all AN patients. Moreover, the 10 AN patients complicated with vestibular dysfunction had normal oculomotor center, no spontaneous nystagmus and caloric test-induced nystagmus response abnormalities. The results were consistent with those reported by Sinha et al.,^12^ suggesting that the vestibular branch of auditory nerve and its dominant structure were also involved. However, due to the slow progression of vestibular neuropathy, the vestibular dysfunction of auditory neurosis patients can be compensated by various compensatory mechanisms, mostly manifested as asymptomatic vestibular dysfunction.^13^

In this study, 23 AN patients were accompanied by other neurological symptoms and one case of progressive fibular muscular dystrophy which belonged to a genetic motor sensory neuropathy I type, myelin type. Another two patients were diagnosed as Friedreich ataxia, belonging to hereditary ataxia Typ-I (spinal type). There was also one patient who received treatment for both lower extremity weakness and severe visual impairment. Visual electrophysiological examination showed bilateral visual conduction dysfunction and Refsum disease. Refsum disease, also known as phytanic acid storage disease, is an autosomal recessive genetic disease, belonging to hereditary motor sensory neuropathy Type-IV. Its three main clinical features include retinal pigment degeneration (night blindness), multiple peripheral nerve damage and cerebellar ataxia. The pathogenesis is that phytanic acid cannot be metabolized or deposited in lipid due to the lack of α-hydroxylase.^14^

## CONCLUSION

In summary, according to characteristic hearing dysfunction, AN may occur in the afferent pathway of acoustic nerve, probably accompanied by the pathological changes of efferent nerve in the olivocochlear system inside the brainstem. Whether the pathogenesis of different auditory neuropathies is the same needs to be further studied.

### Authors’ Contributions

**LJ, JC & XF:** Data collection and analysis.

**JW & ZL:** Study design and manuscript writing.
